# Supplementary material for: Travel time prediction of urban public transportation based on detection of single routes
Source: PLoS One. 2022 Jan 14;17(1):e0262535. doi: 10.1371/journal.pone.0262535 (PMC8759653; doi:10.1371/journal.pone.0262535)
Supplement: S1 File — (DOCX) [file pone.0262535.s001.docx]

Supporting information

**S1. Data processing procedures**

**S1.1 Generates the Pattern offset of the road segment**

**S1.1.1 Data structure**

Name: tops_ride_poset

Output: rideID, pattern_name, pattern_oset

Table: Madmetro.shapes

| Field Name | Field Type |
| --- | --- |
| PATTERN_NAME | VARCHAR2(10) |
| SHAPE_PT_LAT | NUMBER |
| SHAPE_PT_LON | NUMBER |
| SHAPE_PT_SEQUENCE | NUMBER |
| SHAPE_DIST_TRAVELED | NUMBER |

Table: Madmetro.pattern

| Field Name | Field Type |
| --- | --- |
| PATTERN_ID | NUMBER |
| PATTERN_ABBR | VARCHAR2(10) |
| PATTERN_NAME | VARCHAR2(10) |

Table: Madmetro.link_gisidx

| Name | Type |
| --- | --- |
| SERVICE_DAY | VARCHAR2(10) |
| BUS | NUMBER |
| TS | DATE |
| LATITUDE | NUMBER |
| LONGITUDE | NUMBER |
| TRANSACTION_TYPE_ID | NUMBER |
| PASS_SEQUENCE | NUMBER |
| SERVICE_TYPE_ID | NUMBER |
| ROUTE | NUMBER |
| ROUTE_DIRECTION_ID | NUMBER |
| BLOCK | VARCHAR2(10) |
| TRIP_ID | NUMBER |
| OVERLOAD | VARCHAR2(10) |
| EXTRA_BUS | VARCHAR2(10) |
| UPDATE_STEP | NUMBER |
| TIME | NUMBER(10) |

Generated by ArcMap 10, by defining a geo-grid of 10*10 meters, each link is correlated to a sequence of grids indexed by X_IDX, Y_IDX.

| Field Name | Field Type |
| --- | --- |
| LINK_ID | NUMBER |
| X_IDX | NUMBER |
| Y_IDX | NUMBER |

Table: Madmetro.ride_detail

Table: Madmetro.tops_ride_pt

The original Madmetro.ride_detail table + the projected coordinates generated by ArcMap 10 by snapping ride coordinates onto transit network defined by Madmetro.shapes.

| Field Name | Field Type |
| --- | --- |
| SERVICE_DAY | VARCHAR2(10) |
| BUS | NUMBER |
| TS | DATE |
| LATITUDE | NUMBER |
| LONGITUDE | NUMBER |
| TRANSACTION_TYPE_ID | NUMBER |
| PASS_SEQUENCE | NUMBER |
| SERVICE_TYPE_ID | NUMBER |
| ROUTE | NUMBER |
| ROUTE_DIRECTION_ID | NUMBER |
| BLOCK | VARCHAR2(10) |
| TRIP_ID | NUMBER |
| OVERLOAD | VARCHAR2(10) |
| EXTRA_BUS | VARCHAR2(10) |
| UPDATE_STEP | NUMBER |
| RIDE_ID | NUMBER(15) |
| TIMEOFDAY | NUMBER(10) |
| NAD83_X | NUMBER(15,5) |
| NAD83_Y | NUMBER(15,5) |
| X_IDX | NUMBER(15,5) |
| Y_IDX | NUMBER(15,5) |

Table: Madmetro.vehicle_detail

| Name | Type |
| --- | --- |
| CALENDAR_DATE | VARCHAR2(10) |
| BUS | NUMBER |
| DRIVER | NUMBER |
| BLOCK_ABBR | VARCHAR2(10) |
| TRIP_ID | NUMBER |
| RUN_NUM | NUMBER |
| ROUTE_ABBR | VARCHAR2(10) |
| ROUTE_DIRECTION_ID | NUMBER |
| SERVICE_TYPE_ID | NUMBER |
| SCHEDULED_TIME | NUMBER |
| ADHERENCE | NUMBER |
| ACTUAL_ARRIVAL_TIME | NUMBER |
| ACTUAL_DEPARTURE_TIME | NUMBER |
| TIME_POINT_ID | NUMBER |
| GEO_NODE_ID | NUMBER |
| PATTERN_ID | NUMBER |

**S1.1.1 Data processor**

Create or replace view madmetro.v_tops_shape_link_all as

Select "PATTERN_NAME","FROM_LAT","FROM_LON","FROM_SEQ","FROM_OSET","TO_OSET","TO_SEQ","TO_LAT","TO_LON","TO_PATTERN_NAME" from

(Select

pattern_name,

shape_pt_lat from_lat,shape_pt_lon from_lon,shape_pt_sequence from_seq,shape_dist_traveled from_oset,

lead(shape_dist_traveled,1) over (order by pattern_name,shape_pt_sequence) to_oset,

lead(shape_pt_sequence,1) over (order by pattern_name,shape_pt_sequence) to_seq,

lead(shape_pt_lat,1) over (order by pattern_name,shape_pt_sequence) to_lat,

lead(shape_pt_lon,1) over (order by pattern_name,shape_pt_sequence) to_lon,

lead(pattern_name,1) over (order by pattern_name,shape_pt_sequence) to_pattern_name

from **madmetro.shapes**) where pattern_name = to_pattern_name

order by pattern_name,from_oset

Create or replace view madmetro.v_tops_shape_link_unique as

select min(rownum) link_id, from_lat, from_lon, to_lat, to_lon,avg(to_oset-from_oset) link_len

from madmetro.v_tops_shape_link_all

group by from_lat,from_lon,to_lat,to_lon

create or replace view **madmetro.v_tops_shape_pt** as

select rownum shape_pt_id,shape_pt_lat lat,shape_pt_lon lon from

(select distinct shape_pt_lat,shape_pt_lon from **madmetro.shapes**)

Create or replace view madmetro.v_tops_shape_link as

select link_id, f.shape_pt_id from_pt_id, t.shape_pt_id to_pt_id,

f.nad83_x from_x, f.nad83_y from_y, t.nad83_x to_x, t.nad83_y to_y,

link_len

from **madmetro.v_tops_shape_link_unique** v

join **madmetro.tops_shape_pt** f

on v.from_lon = f.lon and v.from_lat = f.lat

join madmetro.**tops_shape_pt** t

on v.to_lon = t.lon and v.to_lat = t.lat

create or replace view **madmetro.v_tops_ride_link** as

select x_idx,y_idx,ride_id,link_id,nad83_x,nad83_y,trip_id

from madmetro.**tops_ride_pt** join **madmetro.link_gisidx**

using(x_idx,y_idx)

Create or replace view madmetro.v_tops_ride_snap as

select ride_id,trip_id,link_id,nad83_x x, nad83_y y, from_x,to_x,from_y,to_y

from **madmetro.tops_ride_link**

join **madmetro.v_tops_shape_link** using(link_id)

Create or replace view madmetro.tops_ride_oset as

select ride_id,trip_id,link_id,sqrt((x-from_x)*(x-from_x)+(y-from_y)*(y-from_y)) link_oset

from **madmetro.v_tops_ride_snap**

create or replace view **madmetro.v_vehicle_trip** as

select trip_id,pattern_id,pattern_name from

(select distinct trip_id,pattern_id from **madmetro.vehicle_detail**

where trip_id is not null) join **madmetro.pattern** using(pattern_id)

Create or replace view madmetro.tops_ride_poset as

select ride_id,trip_id,link_id,pattern_name,link_oset+link_from_oset pattern_oset

from **madmetro.v_tops_ride_oset**

join **madmetro.vehicle_trip** using(trip_id)

join **madmetro.v_tops_metro_shape**

using(link_id,pattern_name)

CREATE OR REPLACE VIEW MADMETRO.V_TOPS_RIDE_PREV_TS AS

select service_day,trip_id,pattern_oset x,timeofday t, pattern_name,

lag(service_day,1) over (order by service_day,trip_id,ts) as

prev_service_day,

lag(trip_id,1) over (order by service_day,trip_id,ts) as prev_trip_id,

lag(pattern_oset,1) over (order by service_day,trip_id,ts) as x0,

lag(ts,1) over (order by service_day,trip_id,ts) as prev_ts,

lag(timeofday,1) over (order by service_day,trip_id,ts) as t0

from **madmetro.tops_ride_poset** a

join **madmetro.tops_ride_detail** b

on (a.ride_id = b.ride_id)

order by service_day,trip_id,ts

CREATE OR REPLACE VIEW MADMETRO.V_TOPS_RIDE_NEXT_TS AS

select service_day,trip_id,pattern_oset x,timeofday t, pattern_name,

lead(service_day,1) over (order by service_day,trip_id,ts) as

next_service_day,

lead(trip_id,1) over (order by service_day,trip_id,ts) as next_trip_id,

lead(pattern_oset,1) over (order by service_day,trip_id,ts) as x1,

lead(ts,1) over (order by service_day,trip_id,ts) as next_ts,

lead(timeofday,1) over (order by service_day,trip_id,ts) as t1

from **madmetro.tops_ride_poset** a

join **madmetro.tops_ride_detail** b

on (a.ride_id = b.ride_id)

order by service_day,trip_id,ts

CREATE OR REPLACE VIEW MADMETRO.V_TOPS_RIDE_BOTH AS

select service_day,trip_id, pattern_name,

case when (service_day <> prev_service_day or trip_id <> prev_trip_id)

then -1

when (x0 is null)

then -1

when (service_day <> next_service_day or trip_id <> next_trip_id)

then 1

when (x1 is null)

then 1

else 0 end strtEndFlag,

round(x) x,t,round(x0) x0,t0,round(x1) x1,t1,

case when t=t0 then 0

when abs(x-x0)<10 then 0

when (service_day <> prev_service_day or trip_id <> prev_trip_id) then 0

else (x-x0)/(t-t0) end u0,

case when t=t1 then 0

when abs(x-x1)<10 then 0

when (service_day <> next_service_day or trip_id <> next_trip_id) then 0

else (x-x1)/(t-t1) end u1

from **madmetro.v_tops_ride_prev_ts**

join **madmetro.v_tops_ride_next_ts**

using (service_day, trip_id,x,t,pattern_name)

order by service_day, trip_id, x, t

--where service_day = prev_service_day and service_day = next_service_day

--and trip_id = prev_trip_id and trip_id = next_trip_id

CREATE OR REPLACE VIEW MADMETRO.V_TOPS_RIDE_FLAG AS

select service_day,trip_id,x,t, pattern_name, u0,u1,

case when u0=0 and u1>0 then 1

when u0 is null and u1>0 then 1

when u1=0 and u0>0 then -1

when u1 is null and u0>0 then -1

when u1>0 and u0>0 then 2

else 0 end flag

**S1.2 Generate stop dwell time(DWT)**

Join with tops_stop_poset

Name: TT_TRIP_STOP

Output: service_day, trip_id, geo_node_id, actual_arrival_time,

actual_departure_time

-------------------------------------------------------------------

create or replace view **madmetro.kt_stop_match_10**

as

select distinct *

from(

select service_day, trip_id, geo_node_id, x, t, a.pattern_name, u0, u1, flag, link_id

from madmetro.v_tops_ride_flag a

join madmetro.tops_stop_poset b

on (a.pattern_name = b.pattern_name and abs(a.x-b.pattern_oset)<10 and flag <> 0)

order by service_day, trip_id, x,t) a

create or replace view **madmetro.kt_TRIP_arrival**

as

select service_day, trip_id, geo_node_id, x, T as actual_arrival_time

from kt_stop_match_10

where flag = -1 or flag = 2

order by service_day, trip_id, x

create or replace view **madmetro.kt_TRIP_departure**

as

select service_day, trip_id, geo_node_id, x, T as actual_departure_time

from **kt_stop_match_10**

where flag = 1 or flag = 2

order by service_day, trip_id, x

create or replace view **madmetro.TT_TRIP_STOP**

as

select a.service_day, a.trip_id, a.geo_node_id, a.x, actual_arrival_time, actual_departure_time

from madmetro.**kt_TRIP_arrival** a

join madmetro.**kt_TRIP_departure** b

on (a.service_day = b.service_day and a.trip_id = b.trip_id and a.geo_node_id = b.geo_node_id)

**S1.3 Generate route travel time(RT)**

Name: TT_TRIP_RUN

Output: service_day, trip_id, start_poset, end_poset,

start_time,end_time,running_time,running_speed

create or replace view **madmetro.kt_trip_run** as

select service_day, trip_id, geo_node_id as start_poset, x as x0, actual_arrival_time as ta0, actual_departure_time as td0,

lead(service_day,1) over (order by service_day,trip_id,x,actual_arrival_time) as end_service_day,

lead(trip_id,1) over (order by service_day,trip_id,x,actual_arrival_time) as end_trip_id,

lead(geo_node_id,1) over (order by service_day,trip_id,x,actual_arrival_time) as end_poset,

lead(x,1) over (order by service_day,trip_id,x,actual_arrival_time) as x1,

lead(actual_arrival_time,1) over (order by service_day,trip_id,x,actual_arrival_time) as ta1,

lead(actual_departure_time,1) over (order by service_day,trip_id,x,actual_arrival_time) as td1

from **madmetro.tt_trip_stop**

create or replace view **madmetro.tt_trip_run** as

select *

from (

select service_day, trip_id, start_poset, end_poset, td0 as start_time, ta1 as end_time,

case when (service_day = end_service_day and trip_id = end_trip_id)

then (ta1-td0) end running_time,

case when (service_day = end_service_day and trip_id = end_trip_id and ta1<>td0)

then (x1-x0)/(ta1-td0) end running_speed

from **madmetro.kt_trip_run**)

where running_speed is not null
