# Supplementary material for: Travel time prediction of urban public transportation based on detection of single routes
Source: PLoS One. 2022 Jan 14;17(1):e0262535. doi: 10.1371/journal.pone.0262535 (PMC8759653; doi:10.1371/journal.pone.0262535)
Supplement: S2 File — (DOCX) [file pone.0262535.s002.docx]

Supporting information

**S2. Data processing results(parts)**

**Table 1. Segmentation of travel time between stops**

| **Date** | **NO. Pattern** | **ID of Stops** | **Arrive time after Segmentation（IC）** | **Departure time after Segmentation（IC）** | **Actual arrive time**  **（AVL）** | **Actual departure time（AVL）** | **Arrive time error（s）** | **Departure time Error（s）** |
| --- | --- | --- | --- | --- | --- | --- | --- | --- |
| 20191001 | 529062 | 1277 | 22105 | 22105 | 22024 | 22073 | 81 | 32 |
| 20191001 | 529225 | 1277 | 32857 | 32915 | 32764 | 32832 | 93 | 83 |
| 20191001 | 529324 | 1277 | 43844 | 43906 | 43828 | 43878 | 16 | 28 |
| 20191001 | 529196 | 1277 | 47179 | 47193 | 47159 | 47223 | 20 | 30 |
| 20191001 | 529465 | 964 | 74031 | 74048 | 74005 | 74203 | 26 | 155 |
| 20191001 | 529281 | 770 | 70009 | 70009 | 70001 | 70067 | 8 | 58 |
| 20191001 | 529400 | 770 | 66241 | 66244 | 66178 | 66197 | 63 | 47 |
| 20191001 | 529062 | 1277 | 22066 | 22076 | 21987 | 22036 | 79 | 40 |
| 20191001 | 529135 | 1277 | 36589 | 36592 | 36511 | 36558 | 78 | 34 |
| 20191001 | 529225 | 964 | 34550 | 34557 | 34529 | 34616 | 21 | 59 |
| 20191001 | 529400 | 3459 | 65799 | 65799 | 65789 | 65821 | 10 | 22 |
| 20191001 | 529400 | 1277 | 65244 | 65258 | 65224 | 65282 | 20 | 24 |
| 20191001 | 529689 | 964 | 41475 | 41519 | 41421 | 41700 | 54 | 181 |
| 20191001 | 529689 | 1277 | 40007 | 40092 | 39984 | 40026 | 23 | 66 |
| 20191001 | 529689 | 1005 | 42066 | 42202 | 41978 | 42288 | 88 | 86 |
| 20191001 | 529689 | 1277 | 40034 | 40035 | 39932 | 40005 | 102 | 30 |
| 20191001 | 529137 | 770 | 84096 | 84099 | 84054 | 84073 | 42 | 26 |
| 20191001 | 529135 | 964 | 38319 | 38346 | 38295 | 38382 | 24 | 36 |
| 20191001 | 529591 | 964 | 30917 | 30917 | 30895 | 30990 | 22 | 73 |
| 20191001 | 529441 | 770 | 77065 | 77156 | 76985 | 77035 | 80 | 121 |
| 20191001 | 529441 | 1277 | 76102 | 76158 | 76074 | 76119 | 28 | 39 |
| 20191001 | 529225 | 1277 | 32901 | 32956 | 32800 | 32872 | 101 | 84 |
| 20191001 | 529371 | 1842 | 79454 | 79454 | 79230 | 79383 | 224 | 71 |
| 20191001 | 529689 | 1005 | 42145 | 42147 | 42000 | 42240 | 145 | 93 |
| 20191001 | 529196 | 770 | 48187 | 48225 | 48193 | 48206 | 6 | 19 |
| 20191001 | 529196 | 1277 | 47245 | 47250 | 47180 | 47220 | 65 | 30 |
| 20191001 | 529324 | 964 | 45226 | 45233 | 45202 | 45613 | 24 | 380 |
| 20191001 | 529324 | 770 | 44616 | 44666 | 44623 | 44636 | 7 | 30 |
| 20191001 | 529371 | 770 | 80632 | 80632 | 80589 | 80636 | 43 | 4 |
| 20191001 | 529371 | 1277 | 79612 | 79612 | 79519 | 79573 | 93 | 39 |
| 20191001 | 529135 | 1277 | 36466 | 36517 | 36446 | 36519 | 20 | 2 |
| 20191001 | 529196 | 1005 | 49346 | 49346 | 49330 | 49520 | 16 | 174 |
| 20191001 | 529196 | 1277 | 47248 | 47248 | 47162 | 47264 | 86 | 16 |
| 20191001 | 529195 | 1277 | 25755 | 25759 | 25638 | 25727 | 117 | 32 |
| 20191001 | 529465 | 1277 | 72415 | 72418 | 72387 | 72440 | 28 | 22 |
| 20191001 | 529441 | 1005 | 77962 | 77968 | 77928 | 77988 | 34 | 20 |
| 20191001 | 529400 | 1277 | 65206 | 65210 | 65178 | 65232 | 28 | 22 |
| 20191002 | 529225 | 1277 | 32802 | 32802 | 32743 | 32835 | 59 | 33 |
| 20191002 | 529196 | 770 | 48229 | 48229 | 48193 | 48207 | 36 | 22 |
| 20191002 | 529371 | 964 | 81055 | 81058 | 81048 | 81114 | 7 | 56 |
| 20191002 | 529689 | 1005 | 42063 | 42182 | 42048 | 42299 | 15 | 117 |
| 20191002 | 529689 | 1277 | 40038 | 40038 | 39942 | 40110 | 96 | 72 |
| 20191002 | 529281 | 770 | 69790 | 69792 | 69761 | 69766 | 29 | 26 |
| 20191002 | 529281 | 3459 | 69284 | 69288 | 69275 | 69301 | 9 | 13 |
| 20191002 | 532843 | 964 | 59734 | 59746 | 59715 | 59924 | 19 | 178 |
| 20191002 | 533534 | 770 | 44927 | 44927 | 44861 | 44887 | 66 | 40 |
| 20191002 | 533534 | 1277 | 43833 | 43902 | 43746 | 43795 | 87 | 107 |
| 20191002 | 529135 | 1277 | 36549 | 36552 | 36464 | 36521 | 85 | 31 |
| 20191002 | 529281 | 3459 | 69269 | 69276 | 69262 | 69292 | 7 | 16 |
| 20191002 | 529195 | 964 | 27160 | 27164 | 27111 | 27708 | 49 | 544 |
| 20191002 | 529592 | 964 | 52613 | 52623 | 52554 | 52953 | 59 | 330 |
| 20191002 | 529592 | 3459 | 51619 | 51679 | 51556 | 51577 | 63 | 102 |
| 20191002 | 529062 | 964 | 23528 | 23528 | 23508 | 23722 | 20 | 194 |
| 20191002 | 529195 | 964 | 27346 | 27346 | 27293 | 27634 | 53 | 288 |
| 20191002 | 529225 | 1277 | 32891 | 32893 | 32782 | 32865 | 109 | 28 |
| 20191002 | 529324 | 1277 | 43579 | 43580 | 43563 | 43628 | 16 | 48 |
| 20191002 | 529135 | 1277 | 36538 | 36559 | 36513 | 36581 | 25 | 22 |
| 20191002 | 529400 | 1277 | 65308 | 65308 | 65228 | 65271 | 80 | 37 |
| 20191002 | 529324 | 770 | 44620 | 44620 | 44613 | 44635 | 7 | 15 |
| 20191002 | 529689 | 1005 | 42008 | 42149 | 41991 | 42224 | 17 | 75 |
| 20191002 | 529281 | 770 | 69858 | 69861 | 69814 | 69834 | 44 | 27 |
| 20191002 | 533534 | 770 | 44681 | 44708 | 44656 | 44725 | 25 | 17 |
| 20191002 | 533534 | 1277 | 43688 | 43688 | 43595 | 43660 | 93 | 28 |
| 20191002 | 532944 | 1277 | 25658 | 25662 | 25553 | 25624 | 105 | 38 |
| 20191002 | 529135 | 1005 | 38649 | 38649 | 38479 | 38634 | 170 | 15 |
| 20191002 | 529689 | 1005 | 42150 | 42153 | 42014 | 42249 | 136 | 96 |
| 20191002 | 529689 | 1277 | 40005 | 40015 | 39899 | 39975 | 106 | 40 |
| 20191002 | 529195 | 964 | 27279 | 27279 | 27190 | 27396 | 89 | 117 |
| 20191002 | 529371 | 3459 | 80296 | 80297 | 80251 | 80256 | 45 | 41 |
| 20191002 | 529225 | 1277 | 32819 | 32826 | 32794 | 32844 | 25 | 18 |
| 20191002 | 529592 | 770 | 51919 | 51919 | 51905 | 51989 | 14 | 70 |
| 20191002 | 529591 | 1277 | 29243 | 29261 | 29207 | 29280 | 36 | 19 |
| 20191002 | 529400 | 770 | 66410 | 66410 | 66357 | 66387 | 53 | 23 |
| 20191002 | 529465 | 964 | 74092 | 74092 | 74027 | 74169 | 65 | 77 |
| 20191002 | 529196 | 1277 | 47347 | 47349 | 47266 | 47319 | 81 | 30 |
| 20191002 | 529281 | 1277 | 68874 | 68874 | 68801 | 68840 | 73 | 34 |
| 20191003 | 529195 | 1277 | 25759 | 25764 | 25652 | 25732 | 107 | 32 |
| 20191003 | 529135 | 1842 | 36059 | 36059 | 35656 | 36042 | 403 | 17 |
| 20191003 | 529591 | 1277 | 29342 | 29342 | 29231 | 29308 | 111 | 34 |
| 20191003 | 529591 | 1842 | 28865 | 28865 | 28511 | 28842 | 354 | 23 |
| 20191003 | 529371 | 770 | 80425 | 80427 | 80413 | 80442 | 12 | 15 |
| 20191003 | 529062 | 1277 | 22070 | 22070 | 21993 | 22036 | 77 | 34 |
| 20191003 | 529441 | 770 | 76892 | 76892 | 76822 | 76867 | 70 | 25 |
| 20191003 | 529400 | 770 | 66157 | 66157 | 66150 | 66208 | 7 | 51 |
| 20191003 | 529324 | 964 | 45634 | 45634 | 45400 | 45681 | 234 | 47 |
| 20191003 | 529591 | 1277 | 29252 | 29257 | 29201 | 29331 | 51 | 74 |
| 20191003 | 529689 | 3459 | 40564 | 40575 | 40550 | 40577 | 14 | 2 |
| 20191003 | 529225 | 964 | 34374 | 34374 | 34352 | 34567 | 22 | 193 |
| 20191003 | 529225 | 1277 | 32790 | 32815 | 32771 | 32833 | 19 | 18 |
| 20191003 | 533271 | 3459 | 62141 | 62141 | 62123 | 62154 | 18 | 13 |
| 20191003 | 533365 | 1277 | 54418 | 54471 | 54397 | 54442 | 21 | 29 |
| 20191003 | 532973 | 1277 | 72405 | 72461 | 72376 | 72432 | 29 | 29 |
| 20191003 | 533560 | 1277 | 43654 | 43657 | 43583 | 43625 | 71 | 32 |
| 20191003 | 533463 | 770 | 51686 | 51745 | 51680 | 51782 | 6 | 37 |
| 20191003 | 533340 | 1277 | 54415 | 54421 | 54397 | 54440 | 18 | 19 |
| 20191003 | 532843 | 770 | 59049 | 59051 | 59043 | 59114 | 6 | 63 |
| 20191003 | 532843 | 1277 | 58018 | 58022 | 57994 | 58042 | 24 | 20 |
| 20191003 | 533534 | 770 | 44790 | 44792 | 44779 | 44804 | 11 | 12 |
| 20191003 | 533145 | 964 | 66987 | 66990 | 66922 | 67009 | 65 | 19 |
| 20191003 | 532748 | 1277 | 29246 | 29254 | 29214 | 29272 | 32 | 18 |
| 20191003 | 532654 | 1277 | 32977 | 33084 | 32957 | 33002 | 20 | 82 |
| 20191003 | 529135 | 964 | 38145 | 38145 | 38125 | 38209 | 20 | 64 |
| 20191003 | 529135 | 1005 | 38681 | 38720 | 38492 | 38642 | 189 | 78 |
| 20191003 | 529689 | 1005 | 42244 | 42247 | 42179 | 42360 | 65 | 113 |
| 20191003 | 529592 | 1005 | 53578 | 53578 | 53562 | 53592 | 16 | 14 |
| 20191003 | 529592 | 1277 | 50890 | 50893 | 50801 | 50860 | 89 | 33 |
| 20191003 | 529591 | 1277 | 29263 | 29278 | 29243 | 29299 | 20 | 21 |
| 20191003 | 529062 | 1277 | 22116 | 22118 | 22045 | 22085 | 71 | 33 |
| 20191003 | 529400 | 1005 | 67348 | 67384 | 67329 | 67386 | 19 | 2 |
| 20191003 | 529441 | 1277 | 76095 | 76095 | 76016 | 76059 | 79 | 36 |
| 20191003 | 529324 | 770 | 44752 | 44752 | 44720 | 44728 | 32 | 24 |
| 20191003 | 529281 | 1277 | 69005 | 69005 | 68914 | 68976 | 91 | 29 |
| 20191003 | 529225 | 964 | 34419 | 34419 | 34398 | 34563 | 21 | 144 |
| 20191003 | 529225 | 770 | 33872 | 33872 | 33862 | 33915 | 10 | 43 |
| 20191003 | 529465 | 770 | 73355 | 73357 | 73293 | 73366 | 62 | 9 |
| 20191003 | 529324 | 770 | 44672 | 44681 | 44612 | 44636 | 60 | 45 |
| 20191003 | 529281 | 770 | 69863 | 69870 | 69835 | 69843 | 28 | 27 |
| 20191003 | 529135 | 1277 | 36428 | 36440 | 36406 | 36469 | 22 | 29 |
| 20191003 | 529592 | 1005 | 52952 | 52952 | 52935 | 52991 | 17 | 39 |
| 20191003 | 529592 | 1277 | 50883 | 50944 | 50804 | 50851 | 79 | 93 |
| 20191003 | 529062 | 964 | 23600 | 23600 | 23576 | 23668 | 24 | 68 |
| 20191003 | 529225 | 1050 | 35330 | 35336 | 35286 | 35294 | 44 | 42 |
| 20191003 | 529225 | 1277 | 32900 | 32904 | 32788 | 32872 | 112 | 32 |
| 20191003 | 529324 | 964 | 45158 | 45164 | 45136 | 45536 | 22 | 372 |
| 20191003 | 529324 | 1277 | 43572 | 43667 | 43553 | 43622 | 19 | 45 |
| 20191003 | 529135 | 1277 | 36423 | 36464 | 36398 | 36479 | 25 | 15 |
| 20191003 | 529441 | 3459 | 76613 | 76615 | 76604 | 76628 | 9 | 13 |
| 20191003 | 529225 | 1277 | 32925 | 32929 | 32801 | 32895 | 124 | 34 |
| 20191003 | 529371 | 770 | 80400 | 80400 | 80392 | 80424 | 8 | 24 |
| 20191003 | 529689 | 1005 | 42209 | 42212 | 42094 | 42322 | 115 | 110 |
| 20191004 | 529689 | 1277 | 40173 | 40224 | 40080 | 40137 | 93 | 87 |
| 20191004 | 529225 | 1277 | 32917 | 32965 | 32818 | 32887 | 99 | 78 |
| 20191004 | 532773 | 770 | 77158 | 77158 | 77065 | 77133 | 93 | 25 |
| 20191004 | 532681 | 1277 | 32832 | 32838 | 32780 | 32868 | 52 | 30 |
| 20191004 | 533340 | 1277 | 54464 | 54464 | 54379 | 54435 | 85 | 29 |
| 20191004 | 533145 | 1277 | 65239 | 65293 | 65218 | 65265 | 21 | 28 |
| 20191004 | 532557 | 770 | 48480 | 48483 | 48396 | 48485 | 84 | 2 |
| 20191004 | 529196 | 1005 | 49230 | 49230 | 49217 | 49453 | 13 | 223 |
| 20191004 | 529281 | 770 | 69926 | 69926 | 69919 | 70004 | 7 | 78 |
| 20191004 | 529135 | 1277 | 36609 | 36613 | 36519 | 36579 | 90 | 34 |
| 20191004 | 529062 | 1277 | 22151 | 22151 | 22063 | 22121 | 88 | 30 |
| 20191004 | 529689 | 1005 | 42182 | 42253 | 42083 | 42323 | 99 | 70 |
| 20191004 | 529196 | 1277 | 47242 | 47275 | 47160 | 47328 | 82 | 53 |
| 20191004 | 529592 | 770 | 51936 | 51944 | 51894 | 51915 | 42 | 29 |
| 20191004 | 529225 | 1277 | 32882 | 32892 | 32781 | 32851 | 101 | 41 |
| 20191004 | 529062 | 1277 | 22006 | 22008 | 21981 | 22034 | 25 | 26 |
| 20191004 | 533560 | 1277 | 43745 | 43747 | 43640 | 43717 | 105 | 30 |
| 20191004 | 532773 | 770 | 77111 | 77111 | 76962 | 77041 | 149 | 70 |
| 20191004 | 533172 | 1277 | 65301 | 65303 | 65214 | 65264 | 87 | 39 |
| 20191004 | 532871 | 1005 | 60211 | 60220 | 60185 | 60238 | 26 | 18 |
| 20191004 | 533436 | 1277 | 50844 | 50849 | 50746 | 50817 | 98 | 32 |
| 20191004 | 529062 | 1277 | 22071 | 22082 | 22048 | 22101 | 23 | 19 |
| 20191004 | 529225 | 1277 | 32855 | 32917 | 32768 | 32830 | 87 | 87 |
| 20191004 | 529135 | 1277 | 36549 | 36596 | 36455 | 36517 | 94 | 79 |
| 20191004 | 529689 | 1005 | 42470 | 42473 | 42327 | 42540 | 143 | 67 |
| 20191004 | 529371 | 770 | 80419 | 80419 | 80408 | 80431 | 11 | 12 |
| 20191004 | 529062 | 964 | 23624 | 23624 | 23575 | 23658 | 49 | 34 |
| 20191004 | 529062 | 770 | 23066 | 23066 | 23047 | 23113 | 19 | 47 |
| 20191004 | 529062 | 1277 | 22125 | 22127 | 22042 | 22092 | 83 | 35 |
| 20191004 | 529400 | 1050 | 67716 | 67719 | 67724 | 67729 | 8 | 10 |
| 20191004 | 529400 | 1277 | 65223 | 65240 | 65195 | 65259 | 28 | 19 |
| 20191004 | 529324 | 1277 | 43686 | 43688 | 43552 | 43662 | 134 | 26 |
| 20191004 | 529281 | 770 | 69914 | 69947 | 69903 | 69926 | 11 | 21 |
| 20191004 | 529400 | 770 | 66317 | 66320 | 66239 | 66294 | 78 | 26 |
| 20191004 | 529196 | 1277 | 47203 | 47253 | 47171 | 47220 | 32 | 33 |
| 20191004 | 529195 | 964 | 27409 | 27409 | 27374 | 27469 | 35 | 60 |
| 20191004 | 529195 | 1277 | 25731 | 25737 | 25644 | 25703 | 87 | 34 |
| 20191004 | 529225 | 964 | 34486 | 34503 | 34466 | 34610 | 20 | 107 |
| 20191004 | 529400 | 964 | 66803 | 66818 | 66783 | 66863 | 20 | 45 |
| 20191004 | 529196 | 1277 | 47200 | 47317 | 47176 | 47275 | 24 | 42 |
| 20191004 | 529689 | 1277 | 40159 | 40159 | 40088 | 40128 | 71 | 31 |

**Table 2. Travel time prediction based on single route detection**

|  | **IC** | **IC** | **IC** | **IC** |  |  |  |  |  |  |  |  |  |  |
| --- | --- | --- | --- | --- | --- | --- | --- | --- | --- | --- | --- | --- | --- | --- |
| **Time** | ***art*3** | ***art*2** | ***art*1** | ***art*(k)** | **average** | **∆3** | **∆2** | **∆1** | **VAR[local]** | ***e(k)*** | ***g*(*k*+1)** | ***a*(*k*+1)** | ***RT_n (i，i_*_+1_*_)_* (*k*+1)** | ***e*(*k*+1)** |
| 2：54：00 | 67 | 65 | 84 | 77 | 71.87 | 25.00 | 49.00 | 144.00 | 72.67 | 0.00 | 0.50 | 0.50 | 80.30 | 36.33 |
| 3：24：00 | 90 | 85 | 89 | 77 | 87.87 | 4.00 | 9.00 | 1.00 | 4.67 | 36.33 | 0.90 | 0.10 | 87.62 | 4.19 |
| 3：54：00 | 103 | 94 | 111 | 94 | 102.54 | 0.11 | 75.11 | 69.44 | 48.22 | 4.19 | 0.52 | 0.48 | 102.72 | 25.11 |
| 4：24：00 | 263 | 108 | 130 | 105 | 166.87 | 9216.00 | 3481.00 | 1369.00 | 4688.67 | 25.11 | 0.50 | 0.50 | 117.40 | 2350.60 |
| 4：54：00 | 116 | 126 | 114 | 94 | 118.54 | 7.11 | 53.78 | 21.78 | 27.56 | 2350.60 | 0.99 | 0.01 | 113.64 | **27.24** |
| **5：24：00** | **102** | **128** | **134** | **124** | **121.18** | **364.94** | **41.17** | **160.95** | **189.02** | **27.24** | **0.53** | **0.47** | **129.21** | **101** |
| 5：54：00 | 136 | 172 | 164 | 128 | 157.29 | 436.39 | 204.78 | 43.30 | 228.15 | 100.86 | 0.59 | 0.41 | 149.13 | 134.73 |
| 6：22：00 | 212 | 148 | 178 | 184 | 179.26 | 1076.93 | 987.43 | 1.94 | 688.77 | 134.73 | 0.54 | 0.46 | 180.60 | 375.06 |
| 6：52：00 | 183 | 171 | 209 | 164 | 187.54 | 21.78 | 277.78 | 455.11 | 251.56 | 375.06 | 0.71 | 0.29 | 195.98 | 179.50 |
| 7：22：00 | 236 | 248 | 236 | 190 | 239.87 | 16.00 | 64.00 | 16.00 | 32.00 | 179.50 | 0.87 | 0.13 | 229.82 | 27.79 |
| 7：52：00 | 187 | 237 | 210 | 240 | 211.05 | 599.76 | 658.95 | 1.39 | 420.03 | 27.79 | 0.52 | 0.48 | 224.39 | 216.74 |
| 8：22：00 | 268 | 198 | 265 | 235 | 243.76 | 607.29 | 2093.67 | 445.77 | 1048.91 | 216.74 | 0.55 | 0.45 | 251.27 | 573.57 |
| 8：50：00 | 188 | 207 | 210 | 233 | 201.66 | 178.04 | 26.39 | 67.35 | 90.59 | 573.57 | 0.88 | 0.12 | 212.63 | 79.72 |
| 9：20：00 | 188 | 269 | 215 | 232 | 223.87 | 1296.00 | 2025.00 | 81.00 | 1134.00 | 79.72 | 0.52 | 0.48 | 223.08 | 586.25 |

**Note：art1：**${art}_{1}\left( k+1 \right)$， is the travel time of bus *n* between stops *i* and *i* +1 during period *k*+1 on the previous day;

**art2：**${art}_{2}\left( k+1 \right)$， is the travel time of bus *n* between stops *i* and *i* +1 during period k+1 on the previous two days;

**art3：**${art}_{3}\left( k+1 \right)$， is the travel time of bus *n* between stops *i* and *i* +1 during period k+1 on the previous three days.

**Table 3. The prediction of bus passenger arrival rate**

| **Time** | ***par*3** | ***par*2** | ***par*1** | ***par*k** | ***aver*** | **∆3** | **∆2** | **∆1** | **VAR[local]** | ***e*(*k*)** | ***g*(*k*+1)** | ***a*(*k*+1)** | ***λ*** | ***e*(*k*+1)** | **Headway** | ***ρ*** | **DWT** |
| --- | --- | --- | --- | --- | --- | --- | --- | --- | --- | --- | --- | --- | --- | --- | --- | --- | --- |
| 2：54：00 | 0.003 | 0.002 | 0.004 | 0.003 | 0.0030 | 0.00000003 | 0.00000055 | 0.00000086 | 0.00000048 | 0.0000000 | 0.50 | 0.50 | 0.004 | 0.0000002 | 1320.00 | 2.50 | 11.92 |
| 3：24：00 | 0.004 | 0.002 | 0.003 | 0.004 | 0.0031 | 0.00000168 | 0.00000086 | 0.00000014 | 0.00000089 | 0.0000002 | 0.56 | 0.44 | 0.004 | 0.0000005 | 1440.00 | 2.50 | 12.64 |
| 3：54：00 | 0.003 | 0.002 | 0.005 | 0.006 | 0.0033 | 0.00000000 | 0.00000278 | 0.00000278 | 0.00000185 | 0.0000005 | 0.56 | 0.44 | 0.005 | 0.0000010 | 2160.00 | 2.50 | 28.32 |
| 4：24：00 | 0.006 | 0.006 | 0.004 | 0.005 | 0.0054 | 0.00000003 | 0.00000055 | 0.00000086 | 0.00000048 | 0.0000010 | 0.76 | 0.24 | 0.005 | 0.0000004 | 1260.00 | 2.50 | 14.42 |
| 4：54：00 | 0.005 | 0.004 | 0.005 | 0.006 | 0.0048 | 0.00000003 | 0.00000014 | 0.00000003 | 0.00000007 | 0.0000004 | 0.86 | 0.14 | 0.005 | **0.00000006** | 2400.00 | 2.50 | 30.46 |
| **5：24：00** | **0.0044** | **0.0039** | **0.0044** | **0.0044** | **0.0043** | **0.00000003** | **0.00000014** | **0.00000003** | **0.00000007** | **0.00000006** | **0.65** | **0.35** | **0.004** | **0.00000004** | **1800.00** | **2.50** | **20.00** |
| 5：54：00 | 0.002 | 0.002 | 0.002 | 0.003 | 0.0020 | 0.00000003 | 0.00000014 | 0.00000003 | 0.00000007 | 0.00000004 | 0.62 | 0.38 | 0.002 | 0.00000004 | 2040.00 | 2.50 | 12.40 |
| 6：22：00 | 0.005 | 0.004 | 0.004 | 0.005 | 0.0044 | 0.00000031 | 0.00000000 | 0.00000031 | 0.00000021 | 0.00000004 | 0.55 | 0.45 | 0.004 | 0.0000001 | 1500.00 | 2.50 | 16.47 |
| 6：52：00 | 0.003 | 0.003 | 0.002 | 0.004 | 0.0028 | 0.00000031 | 0.00000000 | 0.00000031 | 0.00000021 | 0.0000001 | 0.61 | 0.39 | 0.003 | 0.0000001 | 2340.00 | 2.50 | 16.83 |
| 7：22：00 | 0.004 | 0.004 | 0.004 | 0.003 | 0.0041 | 0.00000003 | 0.00000014 | 0.00000003 | 0.00000007 | 0.0000001 | 0.74 | 0.26 | 0.004 | 0.0000001 | 1920.00 | 2.50 | 17.97 |
| 7：52：00 | 0.003 | 0.004 | 0.005 | 0.004 | 0.0041 | 0.00000055 | 0.00000003 | 0.00000086 | 0.00000048 | 0.0000001 | 0.53 | 0.47 | 0.004 | 0.0000003 | 2040.00 | 2.50 | 22.81 |
| 8：22：00 | 0.004 | 0.003 | 0.004 | 0.004 | 0.0037 | 0.00000003 | 0.00000014 | 0.00000003 | 0.00000007 | 0.0000003 | 0.82 | 0.18 | 0.004 | 0.0000001 | 2160.00 | 2.50 | 21.00 |
| 8：50：00 | 0.004 | 0.003 | 0.004 | 0.003 | 0.0037 | 0.00000003 | 0.00000014 | 0.00000003 | 0.00000007 | 0.0000001 | 0.65 | 0.35 | 0.004 | 0.00000004 | 1560.00 | 2.50 | 14.40 |
| 9：20：00 | 0.003 | 0.003 | 0.002 | 0.002 | 0.0028 | 0.00000031 | 0.00000000 | 0.00000031 | 0.00000021 | 0.00000004 | 0.55 | 0.45 | 0.002 | 0.0000001 | 1860.00 | 2.50 | 9.17 |
| 9：50：00 | 0.003 | 0.002 | 0.003 | 0.002 | 0.0026 | 0.00000003 | 0.00000014 | 0.00000003 | 0.00000007 | 0.0000001 | 0.73 | 0.27 | 0.002 | 0.0000000 | 2040.00 | 2.50 | 12.61 |

**Table 4. Predictive, actual travel time data of route 2**

| **Date** | **No. Pattern** | **ID of stop** | **Predictive arrive time**  **（IC）** | **Predictive departure time（IC）** | **Actual arrive time（AVL）** | **Actual departure time（AVL）** | **Arrival time error（s）** | **Departure time error（s）** |
| --- | --- | --- | --- | --- | --- | --- | --- | --- |
| 20191001 | 529326 | 1139 | 48938 | 48938 | 48848 | 48891 | 90 | 47 |
| 20191001 | 529594 | 760 | 57407 | 57407 | 57333 | 57392 | 74 | 15 |
| 20191001 | 529593 | 1431 | 36281 | 36281 | 36264 | 36292 | 17 | 11 |
| 20191001 | 529467 | 1123 | 78053 | 78054 | 77994 | 78010 | 59 | 44 |
| 20191001 | 529594 | 963 | 56791 | 56791 | 56764 | 56841 | 27 | 50 |
| 20191001 | 529594 | 1123 | 56467 | 56521 | 56415 | 56427 | 52 | 94 |
| 20191001 | 529594 | 1139 | 56212 | 56212 | 56176 | 56180 | 36 | 32 |
| 20191001 | 529593 | 963 | 35224 | 35239 | 35204 | 35266 | 20 | 27 |
| 20191001 | 529692 | 1431 | 47217 | 47217 | 47150 | 47178 | 67 | 39 |
| 20191001 | 529692 | 963 | 46138 | 46146 | 46116 | 46178 | 22 | 32 |
| 20191001 | 529372 | 760 | 61126 | 61126 | 61075 | 61115 | 51 | 11 |
| 20191001 | 529692 | 963 | 46138 | 46142 | 46064 | 46122 | 74 | 20 |
| 20191001 | 529692 | 1139 | 45437 | 45493 | 45430 | 45453 | 7 | 40 |
| 20191001 | 531435 | 1123 | 67252 | 67254 | 67242 | 67273 | 10 | 19 |
| 20191001 | 531435 | 1139 | 66960 | 66960 | 66947 | 66991 | 13 | 31 |
| 20191001 | 529401 | 963 | 70989 | 70989 | 70972 | 71414 | 17 | 425 |
| 20191001 | 529593 | 760 | 35735 | 35735 | 35700 | 35781 | 35 | 46 |
| 20191001 | 529226 | 760 | 39262 | 39268 | 39248 | 39316 | 14 | 48 |
| 20191001 | 529226 | 963 | 38803 | 38803 | 38721 | 38906 | 82 | 103 |

| 20191001 | 529326 | 1431 | 50669 | 50674 | 50615 | 50622 | 54 | 52 |
| --- | --- | --- | --- | --- | --- | --- | --- | --- |
| 20191001 | 529326 | 963 | 49540 | 49587 | 49515 | 49700 | 25 | 113 |
| 20191001 | 529372 | 1123 | 60160 | 60160 | 60075 | 60122 | 85 | 38 |
| 20191001 | 529692 | 963 | 46020 | 46082 | 46001 | 46057 | 19 | 25 |
| 20191001 | 529138 | 760 | 42937 | 42937 | 42853 | 42988 | 84 | 51 |
| 20191001 | 529138 | 1139 | 41753 | 41763 | 41694 | 41701 | 59 | 62 |
| 20191001 | 529139 | 1431 | 65119 | 65119 | 65019 | 65129 | 100 | 10 |
| 20191001 | 529594 | 963 | 56954 | 56954 | 56854 | 56925 | 100 | 29 |
| 20191001 | 529594 | 1123 | 56305 | 56448 | 56150 | 56425 | 155 | 23 |
| 20191001 | 529226 | 963 | 38776 | 38776 | 38657 | 38840 | 119 | 64 |
| 20191001 | 529467 | 1123 | 78089 | 78089 | 78058 | 78101 | 31 | 12 |
| 20191001 | 529326 | 760 | 50276 | 50317 | 50200 | 50261 | 76 | 56 |
| 20191001 | 529198 | 760 | 53795 | 53800 | 53704 | 53785 | 91 | 15 |
| 20191001 | 529198 | 1139 | 52709 | 52718 | 52669 | 52675 | 40 | 43 |
| 20191001 | 532974 | 760 | 68057 | 68057 | 67951 | 68103 | 106 | 46 |
| 20191001 | 532876 | 760 | 53975 | 53975 | 53866 | 53953 | 109 | 22 |
| 20191001 | 533148 | 1123 | 60015 | 60019 | 60002 | 60058 | 13 | 39 |
| 20191001 | 532658 | 760 | 28795 | 28797 | 28425 | 28783 | 370 | 14 |
| 20191001 | 533049 | 1123 | 78053 | 78053 | 77947 | 78014 | 106 | 39 |
| 20191001 | 533048 | 760 | 32323 | 32333 | 32301 | 32374 | 22 | 41 |
| 20191001 | 532948 | 1123 | 67180 | 67234 | 67170 | 67197 | 10 | 37 |
| 20191001 | 532752 | 1431 | 72126 | 72128 | 72115 | 72139 | 11 | 11 |
| 20191001 | 532752 | 1123 | 70831 | 70836 | 70735 | 70812 | 96 | 24 |
| 20191002 | 529692 | 1431 | 47294 | 47294 | 47231 | 47258 | 63 | 36 |
| 20191002 | 529692 | 760 | 46754 | 46761 | 46666 | 46742 | 88 | 19 |
| 20191002 | 529372 | 1123 | 60256 | 60297 | 60166 | 60216 | 90 | 81 |
| 20191002 | 529594 | 963 | 56800 | 56800 | 56773 | 56843 | 27 | 43 |
| 20191002 | 529593 | 963 | 35170 | 35189 | 35148 | 35337 | 22 | 148 |
| 20191002 | 529593 | 1123 | 34797 | 34813 | 34785 | 34849 | 12 | 36 |
| 20191002 | 529287 | 1123 | 74507 | 74507 | 74410 | 74470 | 97 | 37 |
| 20191002 | 529226 | 963 | 38791 | 38791 | 38721 | 38842 | 70 | 51 |
| 20191002 | 529326 | 1123 | 49352 | 49353 | 49084 | 49331 | 268 | 22 |
| 20191002 | 529198 | 1431 | 54372 | 54372 | 54313 | 54338 | 59 | 34 |
| 20191002 | 529198 | 760 | 53848 | 53850 | 53810 | 53885 | 38 | 35 |
| 20191002 | 529442 | 760 | 82588 | 82639 | 82515 | 82554 | 73 | 85 |
| 20191002 | 529442 | 1139 | 81423 | 81423 | 81380 | 81385 | 43 | 38 |
| 20191002 | 529372 | 963 | 60652 | 60671 | 60630 | 60704 | 22 | 33 |
| 20191002 | 529198 | 1431 | 54333 | 54513 | 54188 | 54303 | 145 | 210 |
| 20191002 | 529226 | 760 | 39265 | 39265 | 39253 | 39380 | 12 | 115 |
| 20191002 | 529326 | 963 | 49661 | 49714 | 49643 | 49774 | 18 | 60 |
| 20191002 | 529372 | 1123 | 60264 | 60264 | 60253 | 60291 | 11 | 27 |
| 20191002 | 529139 | 1123 | 63708 | 63711 | 63643 | 63672 | 65 | 39 |
| 20191002 | 529139 | 1139 | 63384 | 63410 | 63347 | 63367 | 37 | 43 |
| 20191002 | 529138 | 760 | 42994 | 42997 | 42906 | 42984 | 88 | 13 |
| 20191002 | 529138 | 963 | 42451 | 42481 | 42362 | 42549 | 89 | 68 |
| 20191002 | 529692 | 1431 | 47097 | 47097 | 47033 | 47065 | 64 | 32 |
| 20191002 | 529692 | 1139 | 45472 | 45527 | 45430 | 45437 | 42 | 90 |
| 20191002 | 529326 | 963 | 49533 | 49544 | 49515 | 49767 | 18 | 223 |
| 20191002 | 529372 | 760 | 61020 | 61020 | 60856 | 61008 | 164 | 12 |
| 20191002 | 529372 | 963 | 60404 | 60416 | 60373 | 60443 | 31 | 27 |
| 20191002 | 529139 | 760 | 64503 | 64527 | 64407 | 64593 | 96 | 66 |
| 20191002 | 529594 | 1139 | 56221 | 56221 | 56168 | 56175 | 53 | 46 |
| 20191002 | 529226 | 760 | 39313 | 39313 | 39259 | 39380 | 54 | 67 |
| 20191002 | 529139 | 760 | 64646 | 64646 | 64446 | 64630 | 200 | 16 |
| 20191002 | 529594 | 760 | 57311 | 57322 | 57300 | 57388 | 11 | 66 |
| 20191002 | 529594 | 1123 | 56400 | 56484 | 56391 | 56419 | 9 | 65 |
| 20191002 | 529593 | 963 | 35284 | 35301 | 35149 | 35271 | 135 | 30 |
| 20191002 | 529372 | 963 | 60330 | 60335 | 60298 | 60435 | 32 | 100 |
| 20191002 | 529226 | 1431 | 39833 | 39833 | 39828 | 39849 | 5 | 16 |
| 20191002 | 529226 | 760 | 39303 | 39343 | 39221 | 39330 | 82 | 13 |
| 20191002 | 529226 | 963 | 38750 | 38767 | 38727 | 38843 | 23 | 76 |
| 20191002 | 529326 | 963 | 49672 | 49672 | 49579 | 49764 | 93 | 92 |
| 20191002 | 532587 | 963 | 42531 | 42585 | 42506 | 42572 | 25 | 13 |
| 20191002 | 533075 | 963 | 78373 | 78399 | 78255 | 78442 | 118 | 43 |
| 20191002 | 533467 | 963 | 46078 | 46078 | 46018 | 46066 | 60 | 12 |
| 20191002 | 533467 | 1139 | 45412 | 45414 | 45255 | 45379 | 157 | 35 |
| 20191002 | 533369 | 1123 | 49235 | 49240 | 49223 | 49275 | 12 | 35 |
| 20191002 | 533174 | 760 | 61001 | 61018 | 60989 | 61069 | 12 | 51 |
| 20191002 | 533174 | 963 | 60507 | 60507 | 60381 | 60495 | 126 | 12 |
| 20191002 | 532974 | 1123 | 67239 | 67274 | 67160 | 67185 | 79 | 89 |
| 20191002 | 532876 | 1123 | 52879 | 52907 | 52772 | 52868 | 107 | 39 |
| 20191002 | 529692 | 963 | 46013 | 45965 | 45854 | 45871 | 159 | 94 |
| 20191002 | 529692 | 963 | 45951 | 45965 | 45854 | 45871 | 97 | 94 |
| 20191003 | 529692 | 963 | 46013 | 46013 | 45854 | 45871 | 159 | 142 |
| 20191003 | 529692 | 963 | 45951 | 46013 | 45854 | 45871 | 97 | 142 |
| 20191003 | 529138 | 1431 | 43511 | 43562 | 43500 | 43535 | 11 | 27 |
| 20191003 | 529594 | 1431 | 57867 | 57870 | 57859 | 57880 | 8 | 10 |
| 20191003 | 529401 | 1431 | 72157 | 72157 | 72147 | 72172 | 10 | 15 |
| 20191003 | 529401 | 760 | 71763 | 71763 | 71708 | 71752 | 55 | 11 |
| 20191003 | 529401 | 963 | 71207 | 71207 | 71166 | 71244 | 41 | 37 |
| 20191003 | 529467 | 760 | 78783 | 78796 | 78747 | 78826 | 36 | 30 |
| 20191003 | 529287 | 1123 | 74485 | 74485 | 74474 | 74501 | 11 | 16 |
| 20191003 | 529442 | 760 | 82497 | 82497 | 82346 | 82664 | 151 | 167 |
| 20191003 | 529226 | 1123 | 38564 | 38566 | 38392 | 38544 | 172 | 22 |
| 20191003 | 529326 | 963 | 49747 | 49747 | 49638 | 49771 | 109 | 24 |
| 20191003 | 529139 | 1123 | 63647 | 63663 | 63636 | 63685 | 11 | 22 |
| 20191003 | 529594 | 760 | 57584 | 57586 | 57520 | 57574 | 64 | 12 |
| 20191003 | 529372 | 760 | 60979 | 60983 | 60970 | 61011 | 9 | 28 |
| 20191003 | 529372 | 963 | 60557 | 60561 | 60538 | 60612 | 19 | 51 |
| 20191003 | 529372 | 963 | 60402 | 60402 | 60378 | 60463 | 24 | 61 |
| 20191003 | 529594 | 1123 | 56443 | 56454 | 56383 | 56409 | 60 | 45 |
| 20191003 | 529593 | 1431 | 36291 | 36291 | 36283 | 36305 | 8 | 14 |
| 20191003 | 529593 | 760 | 35802 | 35802 | 35786 | 35861 | 16 | 59 |
| 20191003 | 529593 | 1123 | 34834 | 34879 | 34818 | 34858 | 16 | 21 |
| 20191003 | 529692 | 963 | 46086 | 46086 | 46007 | 46123 | 79 | 37 |
| 20191003 | 529139 | 760 | 64485 | 64490 | 64478 | 64588 | 7 | 98 |
| 20191003 | 529139 | 1123 | 63644 | 63644 | 63620 | 63658 | 24 | 14 |
| 20191003 | 529138 | 1123 | 41939 | 41942 | 41928 | 41973 | 11 | 31 |
| 20191003 | 531435 | 760 | 67991 | 67991 | 67937 | 68017 | 54 | 26 |
| 20191003 | 531435 | 963 | 67621 | 67621 | 67608 | 67642 | 13 | 21 |
| 20191003 | 529226 | 760 | 39233 | 39302 | 39223 | 39380 | 10 | 78 |
| 20191003 | 529226 | 963 | 38854 | 38854 | 38787 | 38843 | 67 | 11 |
| 20191003 | 529226 | 1123 | 38512 | 38512 | 38494 | 38534 | 18 | 22 |
| 20191003 | 532587 | 963 | 42390 | 42399 | 42374 | 42490 | 16 | 91 |
| 20191003 | 533562 | 963 | 38822 | 38834 | 38800 | 39107 | 22 | 273 |
| 20191003 | 533562 | 1123 | 38516 | 38519 | 38498 | 38558 | 18 | 39 |
| 20191003 | 532683 | 1123 | 27709 | 27709 | 27610 | 27674 | 99 | 35 |
| 20191003 | 533467 | 1139 | 45416 | 45416 | 45284 | 45370 | 132 | 46 |
| 20191003 | 532948 | 1123 | 67210 | 67210 | 67149 | 67174 | 61 | 36 |
| 20191003 | 533537 | 760 | 39324 | 39326 | 39312 | 39382 | 12 | 56 |
| 20191003 | 533048 | 1123 | 31294 | 31294 | 31212 | 31276 | 82 | 18 |
| 20191003 | 533048 | 1139 | 30931 | 30933 | 30886 | 30892 | 45 | 41 |
| 20191003 | 532752 | 760 | 71753 | 71792 | 71702 | 71784 | 51 | 8 |
| 20191003 | 532752 | 963 | 71144 | 71262 | 71098 | 71231 | 46 | 31 |
| 20191003 | 532658 | 1123 | 27795 | 27814 | 27708 | 27755 | 87 | 59 |
| 20191003 | 529138 | 1123 | 42144 | 42144 | 42066 | 42097 | 78 | 47 |
| 20191003 | 529287 | 963 | 74813 | 74820 | 74740 | 74802 | 73 | 18 |
| 20191003 | 529372 | 963 | 60321 | 60321 | 60300 | 60376 | 21 | 55 |
| 20191003 | 529692 | 1431 | 47201 | 47201 | 47131 | 47153 | 70 | 48 |
| 20191003 | 529692 | 1123 | 45693 | 45710 | 45679 | 45731 | 14 | 21 |
| 20191003 | 529226 | 963 | 38739 | 38750 | 38724 | 38843 | 15 | 93 |
| 20191003 | 529326 | 1123 | 49325 | 49328 | 49107 | 49301 | 218 | 27 |
| 20191003 | 529372 | 1431 | 61471 | 61471 | 61416 | 61439 | 55 | 32 |
| 20191004 | 529372 | 963 | 60399 | 60399 | 60379 | 60441 | 20 | 42 |
| 20191004 | 529372 | 1123 | 60154 | 60194 | 60064 | 60104 | 90 | 90 |
| 20191004 | 529138 | 760 | 43036 | 43094 | 42979 | 43082 | 57 | 12 |
| 20191004 | 529692 | 963 | 46077 | 46210 | 46054 | 46177 | 23 | 33 |
| 20191004 | 529594 | 760 | 57398 | 57398 | 57296 | 57387 | 102 | 11 |
| 20191004 | 529226 | 760 | 39327 | 39327 | 39256 | 39312 | 71 | 15 |
| 20191004 | 529226 | 963 | 38732 | 38884 | 38715 | 38832 | 17 | 52 |
| 20191004 | 529326 | 760 | 50269 | 50269 | 50195 | 50258 | 74 | 11 |
| 20191004 | 529139 | 963 | 64026 | 64034 | 63993 | 64117 | 33 | 83 |
| 20191004 | 529138 | 1431 | 43512 | 43512 | 43471 | 43475 | 41 | 37 |
| 20191004 | 529138 | 760 | 42995 | 43028 | 42980 | 43060 | 15 | 32 |
| 20191004 | 529138 | 963 | 42387 | 42431 | 42355 | 42607 | 32 | 176 |
| 20191004 | 529594 | 1431 | 57912 | 57912 | 57861 | 57882 | 51 | 30 |
| 20191004 | 529594 | 1123 | 56420 | 56457 | 56410 | 56436 | 10 | 21 |
| 20191004 | 529593 | 1139 | 34591 | 34591 | 34517 | 34557 | 74 | 34 |
| 20191004 | 529198 | 1431 | 54308 | 54308 | 54236 | 54263 | 72 | 45 |
| 20191004 | 532460 | 760 | 64798 | 64798 | 64740 | 64821 | 58 | 23 |
| 20191004 | 532460 | 1123 | 63868 | 63887 | 63853 | 63902 | 15 | 15 |
| 20191004 | 532752 | 760 | 71731 | 71733 | 71619 | 71721 | 112 | 12 |
| 20191004 | 533440 | 1431 | 47092 | 47100 | 47083 | 47110 | 9 | 10 |
| 20191004 | 533440 | 1123 | 45677 | 45681 | 45522 | 45657 | 155 | 24 |
| 20191004 | 532848 | 760 | 54078 | 54123 | 54023 | 54110 | 55 | 13 |
| 20191004 | 533247 | 1123 | 56459 | 56459 | 56392 | 56419 | 67 | 40 |
| 20191004 | 533247 | 1139 | 56216 | 56218 | 56117 | 56181 | 99 | 37 |
| 20191004 | 529138 | 1431 | 43541 | 43541 | 43491 | 43514 | 50 | 27 |
| 20191004 | 529139 | 1123 | 63815 | 63823 | 63743 | 63780 | 72 | 43 |
| 20191004 | 529198 | 1123 | 52868 | 52980 | 52852 | 52887 | 16 | 93 |
| 20191004 | 531435 | 760 | 68031 | 68034 | 67946 | 68020 | 85 | 14 |
| 20191004 | 529372 | 1123 | 60086 | 60086 | 60074 | 60106 | 12 | 20 |
| 20191004 | 529226 | 760 | 39264 | 39272 | 39253 | 39378 | 11 | 106 |
| 20191004 | 529226 | 963 | 38796 | 38796 | 38722 | 38851 | 74 | 55 |
| 20191004 | 529139 | 760 | 64630 | 64658 | 64592 | 64647 | 38 | 11 |
| 20191004 | 529139 | 963 | 64028 | 64030 | 64010 | 64069 | 18 | 39 |
| 20191004 | 529692 | 760 | 46548 | 46548 | 46502 | 46535 | 46 | 13 |
| 20191004 | 529692 | 963 | 45997 | 45997 | 45933 | 46063 | 64 | 66 |
| 20191004 | 529287 | 963 | 74580 | 74580 | 74547 | 74858 | 33 | 278 |
| 20191004 | 529372 | 963 | 60949 | 60949 | 60865 | 60931 | 84 | 18 |
| 20191004 | 529372 | 1123 | 60421 | 60472 | 60405 | 60451 | 16 | 21 |
| 20191004 | 529594 | 963 | 56803 | 56803 | 56777 | 56845 | 26 | 42 |
| 20191004 | 529326 | 1431 | 50810 | 50860 | 50797 | 50821 | 13 | 39 |
| 20191004 | 529326 | 1123 | 49166 | 49326 | 49152 | 49308 | 14 | 18 |
| 20191004 | 529326 | 1139 | 48963 | 48977 | 48910 | 48931 | 53 | 46 |
| 20191004 | 529198 | 1431 | 54343 | 54350 | 54291 | 54313 | 52 | 37 |
| 20191004 | 529198 | 760 | 53806 | 53808 | 53737 | 53794 | 69 | 14 |
| 20191004 | 529138 | 760 | 43151 | 43157 | 43067 | 43140 | 84 | 17 |
| 20191004 | 529372 | 963 | 60403 | 60403 | 60385 | 60463 | 18 | 60 |
| 20191004 | 531435 | 963 | 67557 | 67674 | 67529 | 67658 | 28 | 16 |
| 20191004 | 529138 | 1123 | 42022 | 42022 | 41969 | 41985 | 53 | 37 |
| 20191004 | 529594 | 1431 | 58050 | 58050 | 57783 | 58020 | 267 | 30 |
| 20191004 | 529593 | 760 | 35751 | 35764 | 35742 | 35796 | 9 | 32 |
| 20191004 | 529198 | 760 | 53872 | 53872 | 53799 | 53854 | 73 | 18 |
| 20191004 | 529198 | 963 | 53183 | 53246 | 53155 | 53278 | 28 | 32 |
| 20191004 | 529198 | 1139 | 52661 | 52661 | 52620 | 52627 | 41 | 34 |
| 20191004 | 529401 | 963 | 71125 | 71169 | 71104 | 71159 | 21 | 10 |
| 20191004 | 529401 | 1123 | 70861 | 70861 | 70794 | 70823 | 67 | 38 |
| 20191004 | 529226 | 1123 | 38575 | 38575 | 38493 | 38539 | 82 | 36 |
| 20191004 | 529594 | 760 | 57363 | 57363 | 57336 | 57401 | 27 | 38 |
| 20191004 | 529326 | 963 | 49649 | 49649 | 49576 | 49703 | 73 | 54 |
| 20191004 | 529226 | 963 | 38802 | 38811 | 38789 | 38845 | 13 | 34 |
| 20191004 | 529372 | 963 | 60560 | 60566 | 60541 | 60598 | 19 | 32 |
| 20191004 | 529372 | 1123 | 60196 | 60198 | 60183 | 60223 | 13 | 25 |
| 20191004 | 529692 | 1139 | 45337 | 45337 | 45287 | 45323 | 50 | 14 |
| 20191004 | 529326 | 963 | 49696 | 49696 | 49637 | 49764 | 59 | 68 |
| 20191004 | 529326 | 1123 | 49128 | 49133 | 49110 | 49284 | 18 | 151 |
| 20191004 | 531435 | 963 | 67585 | 67592 | 67521 | 67571 | 64 | 21 |
| 20191004 | 529287 | 963 | 74785 | 74785 | 74752 | 74820 | 33 | 35 |
| 20191004 | 529692 | 1123 | 45773 | 45777 | 45677 | 45732 | 96 | 45 |
| 20191004 | 529198 | 1123 | 52793 | 52798 | 52780 | 52814 | 13 | 16 |
| 20191004 | 531435 | 1123 | 67119 | 67119 | 67100 | 67143 | 19 | 24 |
| 20191004 | 529226 | 760 | 39327 | 39333 | 39259 | 39317 | 68 | 16 |
| 20191004 | 529139 | 760 | 64609 | 64609 | 64462 | 64597 | 147 | 12 |
| 20191004 | 529138 | 963 | 42629 | 42638 | 42604 | 42675 | 25 | 37 |
| 20191004 | 531435 | 760 | 67961 | 68035 | 67851 | 68017 | 110 | 18 |
| 20191004 | 529226 | 1123 | 38432 | 38494 | 38357 | 38390 | 75 | 104 |
| 20191004 | 529594 | 963 | 56799 | 56842 | 56762 | 56905 | 37 | 63 |
| 20191004 | 529326 | 963 | 49586 | 49601 | 49563 | 49685 | 23 | 84 |
| 20191004 | 529326 | 1123 | 49315 | 49315 | 49113 | 49329 | 202 | 14 |
| 20191004 | 529326 | 1139 | 48940 | 48940 | 48896 | 48911 | 44 | 29 |
| 20191004 | 529326 | 760 | 50193 | 50193 | 50107 | 50185 | 86 | 8 |
| 20191004 | 529372 | 760 | 60970 | 61028 | 60960 | 61011 | 10 | 17 |
| 20191004 | 529287 | 760 | 75389 | 75392 | 75299 | 75379 | 90 | 13 |
| 20191004 | 531435 | 963 | 67828 | 67834 | 67769 | 67818 | 59 | 16 |
| 20191004 | 529226 | 760 | 39632 | 39635 | 39549 | 39621 | 83 | 14 |
| 20191004 | 529226 | 963 | 38929 | 38953 | 38906 | 39024 | 23 | 71 |
| 20191004 | 529226 | 1123 | 38553 | 38558 | 38453 | 38530 | 100 | 28 |
| 20191004 | 529326 | 1123 | 49370 | 49370 | 49077 | 49332 | 293 | 38 |
| 20191004 | 529593 | 963 | 35306 | 35339 | 35261 | 35331 | 45 | 8 |
| 20191004 | 531435 | 1123 | 67373 | 67373 | 67304 | 67342 | 69 | 31 |
| 20191004 | 529138 | 1123 | 41907 | 41907 | 41892 | 41929 | 15 | 22 |
| 20191004 | 529198 | 963 | 53156 | 53168 | 53131 | 53323 | 25 | 155 |
| 20191004 | 529198 | 1123 | 52794 | 52794 | 52776 | 52835 | 18 | 41 |
| 20191004 | 529692 | 1431 | 47167 | 47170 | 47105 | 47135 | 62 | 35 |
| 20191004 | 529198 | 760 | 53691 | 53691 | 53636 | 53733 | 55 | 42 |
