# Supplementary material for: Travel time prediction of urban public transportation based on detection of single routes
Source: PLoS One. 2022 Jan 14;17(1):e0262535. doi: 10.1371/journal.pone.0262535 (PMC8759653; doi:10.1371/journal.pone.0262535)
Supplement: S3 File — (DOCX) [file pone.0262535.s003.docx]

Supporting information

**S3. The layout of Madison transit network**

**Fig 1. Transit network of Madison**

| **** |
| --- |
| **Fig 1. Transit network of Madison** |

**Fig 2. Primary data of Madison’s transit network based on ArcMap**

| **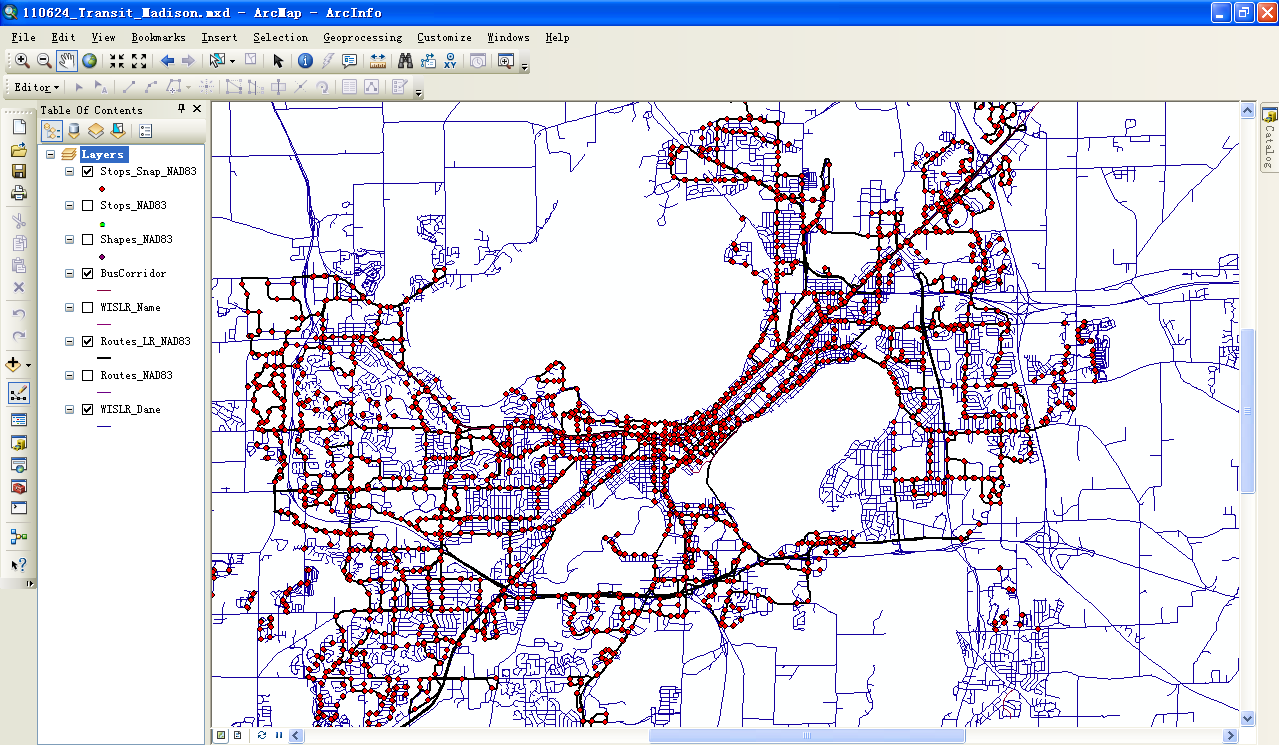** |
| --- |
| **Fig 2. Primary data of Madison’s transit network based on ArcMap** |

**Fig 3. The location of road segment in case**

| **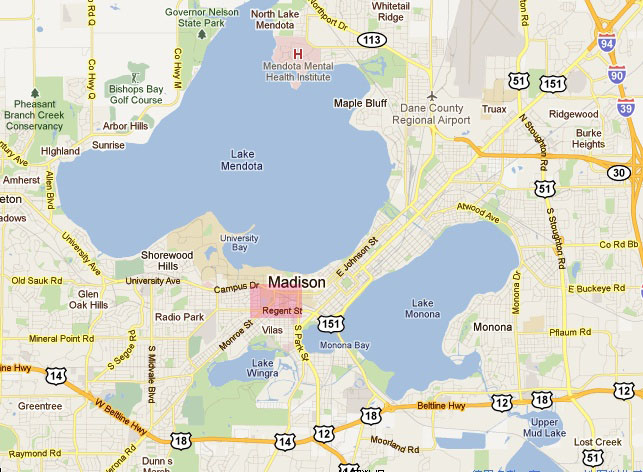** |
| --- |
| **Fig 3. The location of road segment in case** |

| 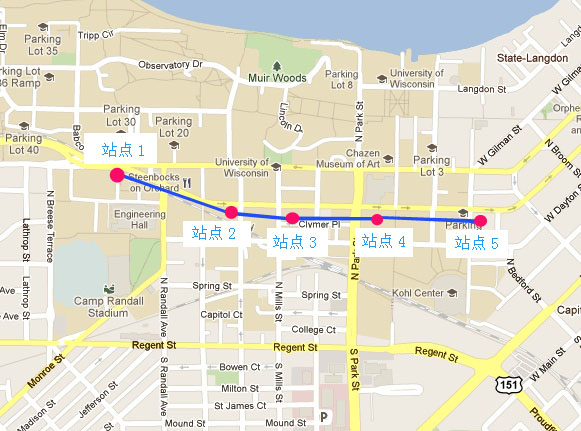 Stop1  Stop2  Stop3  Stop4  Stop5 |
| --- |
| **Fig 4. Road segment in case** |

**Fig 4. Road segment in case**
