# Supplementary material for: Travel time prediction of urban public transportation based on detection of single routes
Source: PLoS One. 2022 Jan 14;17(1):e0262535. doi: 10.1371/journal.pone.0262535 (PMC8759653; doi:10.1371/journal.pone.0262535)
Supplement: Attachment — Submitted filename: 2 Response to Academic Editor.docx [file pone.0262535.s004.docx]

**Response to Academic Editor**

**1. Please ensure that your manuscript meets PLOS ONE's style requirements, including those for file naming.**

**Response:** Ensure that all formats meet the PLOS ONE's style requirements, including those for file naming before the manuscript is submitted

**2. Please provide further details in your methods section of the source of the dataset, how it was collected, and how it can be accessed by other researchers seeking to reproduce your study**

**Response:** The content of the data section has been readjusted as requested. Other researchers can refer to the supporting documentation for details.

**3.We note that *Figure 3* in your submission contains satellite images that may be copyrighted. All PLOS content is published under the Creative Commons Attribution License (CC BY 4.0), which means that the manuscript, images, and Supporting Information files will be freely available online, and any third party is permitted to access, download, copy, distribute, and use these materials in any way, even commercially, with proper attribution. For these reasons, we cannot publish previously copyrighted maps or satellite images created using proprietary data, such as Google software (Google Maps, Street View, and Earth). For more information, see our copyright guidelines: http://journals.plos.org/plosone/s/licenses-and-copyright.
 Response:** I removed Figure 3 in my manuscript.

**4. We suggest you thoroughly copyedit your manuscript for language usage, spelling, and grammar. If you do not know anyone who can help you do this, you may wish to consider employing a professional, scientific editing service. 
Response:** I did thoroughly copyedit my manuscript for language usage, spelling, and grammar. Furthermore, I asked Dr. Les to adjust and modify the syntax error of the manuscript again and polish it in detail. The resume of Mr. Les is attached, which is enough to prove that he is capable of revising academic papers.

***the information of my colleague who edited my manuscript***

**NAME:** Les Lauber（https://www.LinkedIn.com/in/Les-Lauber-ab481/）

Training Specialist focusing on Performance Improvement, Human Resources, and Organizational Development

**Company:** [Professional Development Specialist, Kansas Public Employees Retirement Syst (Full-time).](https://www.linkedin.com/search/results/all/?keywords=Kansas%20Public%20Employees%20Retirement%20Syst)

**Email:** leslauber@hotmail.com

**Phone:** +1 785 219 6157

**5. While revising your submission, please upload your figure files to the Preflight Analysis and Conversion Engine (PACE) digital diagnostic tool,** [**https://pacev2.apexcovantage.com/**](https://pacev2.apexcovantage.com/)**. PACE helps ensure that figures meet PLOS requirements.**

**Response:** According to PLOS ONE's requirements, I have converted all pictures 1, 2, and 3 in the manuscript with professional tools, and Picture 4 is the flow chart generated by Word software. Images 5, 6, 7, and 8 are figures generated directly by the Excel spreadsheet.
