# Supplementary material for: Travel time prediction of urban public transportation based on detection of single routes
Source: PLoS One. 2022 Jan 14;17(1):e0262535. doi: 10.1371/journal.pone.0262535 (PMC8759653; doi:10.1371/journal.pone.0262535)
Supplement: Attachment — Submitted filename: 1 Response to Reviewers.docx [file pone.0262535.s005.docx]

**Response to Reviewers**

**Reviewer #1:**

**The reviewer still has the following comments:**

1. **In the abstract, there is a syntax error in 'Its greater time-variance and uncertainty make predictions for short travel times (≤35min) more subject to influence by random factors'. The 'to influence' should be 'to be influenced'.
   Response:** Thank you very much for your professionalism and carefulness in pointing out the spelling mistakes in the paper. All the errors have been corrected before submitting them to you for review again.
2. **Syntax error. 'It requires higher precision than makes long-term predictions and is more complicated.'
   Response:** Thank you very much for your professionalism and carefulness in pointing out the spelling mistakes in the paper. All the errors have been corrected before submitting them to you for review again.
3. **Syntax error. 'The Kalman filter model has a higher precision in one-step-ahead prediction and can significantly automate massive data calculations to improve prediction accuracy.'**

**Response:** Thank you very much for your professionalism and carefulness in pointing out the spelling mistakes in the paper. All the errors have been corrected before submitting them to you for review again.

4. **The writing of the abstract is hard for readers to understand the background and motivation of this study.**

**Response:** Thank you very much for your criticism and suggestions. I rewrote and adjusted that Section to ensure it was clear and complete.

5. **Syntax error. 'it is essential to study the technology of travel time prediction to more fully meet the data characteristics of urban public transit and improve its applicability.'**

**Response:** Thank you very much for your professionalism and carefulness in pointing out the spelling mistakes in the paper. All the errors have been corrected before submitting them to you for review again.

6. **The flow in Section II confuses readers a lot. In Section II. B, 'While the models described in this Section can solve the bus-to-station prediction problem to some degree, the influence factors these models considered are one-sided. SVM relies too much on kernel tricks to achieve predictions on a large scale. GPS overemphasizes the current state of the bus, degrading the prediction accuracy as the predicted distance increases. The PF considers only the time of the bus to the stop and ignores the spatial effect of the bus. The input used in the NN network is too one-sided and does not consider the comprehensive effect of time and space characteristics.'
   Response:** Thank you for your suggestions. This content has been revised to ensure the integrity and clarity of this part of the content.
7. **Why does the summary of the four models occur before describing the SVM and PF? Would you please move to Section II.E?
   Response:** Thank you for your suggestions. This content has been revised to ensure the integrity and clarity of this part of the content.
8. **Section II describes the advantage of the Kalman Filter over the other four models in the travel time prediction. However, the research gaps and motivations on choosing the Kalman Filter to solve the problem in this study are still unclear. Would you please add more description about limitations of existing studies in travel time prediction with the Kalman Filter?
   Response:** Thank you for your suggestions. The content of more description about limitations of existing studies in travel time prediction with the Kalman Filter has been revised
9. **The research background, academic research gaps, motivations, potential real-world contributions, and renovations of this paper are still unclear by the end of Section II. Would you please state the background, research gaps, motivations, and contributions in Section I&II?**

**Response:** Thank you for your suggestions. The content of background, research gaps, motivations, and contributions have been revised in the Section I&II

10. **The English language and flow through this paper still need to be well organized.**

**Response:** Thank you very much for your criticism and suggestions. In addition to my own readjustment of the logical structure and wording of the text, I also asked professionals to help me polish the paper to ensure its readability.

**Reviewer #2:**

**The authors work out all the comments. And this edition of the manuscript is appropriate for publishing.**

**Response:** Thank you very much for your criticism and suggestions, which enables me to consider the structure of the whole paper more logically and clearly in future writing. Thank you very much for your reply and affirmation, which gives me the confidence to continue writing academic papers in the future. At the same time, based on the editor's advice, I adjusted the contents of the literature part and made the latest adjustments in grammar and diction.

**Reviewer #3:**

**In this revised version of the manuscript, the author has considered all the points in my previous comments. Therefore, in my opinion, this new version of the manuscript can be accepted for publication.**

**Response:** Thank you very much for your criticism and suggestions, which enables me to consider the structure of the whole paper more logically and clearly in future writing. Thank you very much for your reply and affirmation, which gives me the confidence to continue writing academic papers in the future. At the same time, based on the editor's advice, I adjusted the contents of the literature part and made the latest adjustments in grammar and diction.
